# Urban Parks as Perceived by City Residents with Mobility Difficulties: A Qualitative Study with In-Depth Interviews

**DOI:** 10.3390/ijerph19042018

**Published:** 2022-02-11

**Authors:** Magdalena Wojnowska-Heciak, Marzena Suchocka, Magdalena Błaszczyk, Magdalena Muszyńska

**Affiliations:** Department of Landscape Architecture, Institute of Environmental Engineering, Warsaw University of Life Sciences—SGGW, 02-776 Warsaw, Poland; magdalena_blaszczyk@sggw.edu.pl (M.B.); magdalena.muszynska@interia.pl (M.M.)

**Keywords:** urban parks, accessibility, people with mobility difficulties, pavement surface, user perspective

## Abstract

Urban green spaces make an invaluable contribution to the health and well-being of all city residents. Therefore, urban park quality and accessibility are crucial factors in stimulating physical and mental health benefits. This study aimed to assess the quality of urban parks and their accessibility as reported by people with mobility difficulties (seniors, blind and partially sighted people). Four key features of a place (accessibility and linkages, comfort and image, uses and activities and sociability) were considered in an in-depth-interviews (IDI) and “walk-and-talk” interviews. Study results indicate a problem of accessibility of urban parks for people with mobility difficulties (uneven gravel surfaces). However, non-physical aspects of park visits (social activities, cultural events, place branding) were reported as essential factors in explaining the motivation for park visits. Despite individual preferences, experience or reported difficulties, all respondents’ attitudes towards park trips were positive. Therefore, we assume that accessibility is more than just physical comfort. Cultural and social activities play an important role in motivating people with a disability to visit a park.

## 1. Introduction

### 1.1. Sensitive Detectors of Public Space Accessibility

People who encounter mobility difficulties, especially people with a physical disability, are the most sensitive detectors of urban fabric that includes park quality and accessibility [1,2,3,4]. Disability is a complex phenomenon, reflecting an interaction between features of a person’s body and those of the society in which they live. Mobility impairments stem from various causes: a congenital anomaly, impairments caused by disease (e.g., poliomyelitis, bone tuberculosis) or other causes (e.g., cerebral palsy, amputations and fractures or burns that cause contractures) [5]. Different kinds of mobility disability occur with ageing. Mobility issues do not prevent people from travelling using a wheelchair, a power scooter or lifts and slings, crutches, tricycles and bicycles [6]. If the disability is linked to ageing, special walkers are used. Blind or partially sighted people comprise another group. The term blindness is used for complete or nearly complete vision loss [7,8]. Visual impairment may cause difficulties with normal daily activities such as driving, reading, socializing and walking. Many people with severe visual impairments can travel independently, using a range of tools and techniques, such as a white cane with a red tip or a lighter identification (ID) cane, employ guide dogs or Global Positioning System (GPS) devices. Some blind people are skilled at echolocating silent objects simply by producing mouth clicks and listening to the returning echoes [9]. However, sometimes the best solution to travel is to be assisted by another person. Interestingly, able-bodied parents pushing strollers are exposed to a comparable stress due to physical barriers [10,11].

### 1.2. Benefits of Urban Parks to Everyone

Urban parks are parks within or adjacent to urban built-up areas, which have the maximum human interference with the natural environment and the highest level of accessibility for populations [12]. This type of open space offers a variety of economic, environmental and social function values [13,14,15,16] that provide important space-filling elements in the form and layout of cities [17,18,19]. The potential impacts of urban parks on public health have long been recognized in the literature [20,21,22,23,24].

Proximity to parks is a key factor to simulate the physical activity among city residents [16,25,26,27]. Physical activity brings a variety of physiological and psychosocial benefits [28,29,30,31,32,33,34,35,36]. Although their environmental values and behaviour are similar to those of the mainstream society [37], individuals with physical impairments visit green spaces less frequently [38]. Research indicates that people with a disability encounter physical and social barriers, which may lead to feelings of exclusion and outsideness [39,40].

### 1.3. Physical Features of Accessible Public Space

Although the binding documents [40,41,42,43,44,45] address public space accessibility in terms of physical infrastructure and amenities adapted to the needs of people with mobility difficulties, there is still much more to be done in this area. Those provisions specify the dimensions and parameters of passageways, places to rest, ramps, stairs, finishing materials for walls and surfaces (stability, anti-slip), solutions for surface details, windows, doors, entrances, space fittings, restrooms, fences, gates, wickets, lighting and rooms.

Social studies confirm the need to implement above mentioned solutions and the positive role of adapted physical infrastructure in increasing the willingness to visit public spaces. In large open spaces, the route must be guided using natural path markers or distinct boundaries between materials and plants, grass, paving, edges and different orientation types [46]. Accessible restrooms, restaurants and information system (in Braille and other non-text means of communication) should be easily reached from the trail and any other part of the park [46]. Some studies suggest that even the details of the pavement surface play a crucial role. The optimal sidewalk pattern for the pavers is a 90° herringbone pattern, preferred over the 45° pattern. Vibrations experienced while traversing pavement surfaces, with frequent and wide joints in-between pavers, may produce considerable pain or have other adverse effects on the health of individuals using wheelchairs [47]. People with mobility disabilities and visual impairments, older adults and pregnant women also find steps to be a barrier [46]. In addition to physical characteristics, non-physical elements equally determine the urban park quality.

### 1.4. Non-Physical Features of Accessible Public Space

According to the literature, people with a disability have a strong desire to visit natural areas and participate in social activities [46]. Frequent use of green spaces by Danes [48] supports the idea about the importance of easy access to parks, indicating that people with mobility difficulties visit green spaces at least once a week. People are drawn to sites that give them a choice of places to sit so that they can be either in or out of the sun at various times of day or year [45,49]. They choose those forms of recreation that do not involve excessive financial inputs; therefore, visiting an urban park is within their scope of interest. Availability and accessibility have the most decisive impact on their leisure activities. The level of activity depends chiefly on individual preferences and factors [50] such as efforts to overcome self-doubt, redefine oneself and build self-confidence [51]. Having a reason to come to a place (e.g., outdoor fitness equipment, nice views, organized social events) is an effective motivator to leave the house and visit a public space [45].

The non-physical elements affecting the perception of the park’s quality include, among other things, the distinctiveness of the place. For residents, a positive attitude (i.e., place satisfaction, identification, attachment) and recommendation (i.e., positive word-of-mouth) increase the interest in the park. Of great importance is the internal perception of accessibility, sometimes having a stronger influence on park visits than the real obstacles in the built environment [52]. Another significant element that impacts the willingness to visit a public space by people with a disability is that they prefer to be perceived as all other people because the way they treated is as important to them as physical access [46]. The most difficult but the most crucial quality for a place to achieve is sociability. A successful place is a favourite spot for people to meet friends, greet their neighbours and feel comfortable interacting with strangers [45]. The possibility of inviting friends and relatives influences the attachment to a place and hence, visits [53].

People with physical motion constraints have a particular need to have access to sports and recreational spaces to improve their health. They need parks in developing social interactions and build the general spirit [54]. Safety refers not only to the built environment. Perceived safety is an important factor that may attract more visitors or discourage many potential visitors from using and enjoying available public open spaces. Urban parks in central locations of cities are perceived as safer if they are relatively small, manageable, well-maintained and include various activities [55].

### 1.5. Aim

The objective of this study is to deepen the knowledge about park accessibility and infrastructure characteristics that may affect the frequency of visits of people with a mobility difficulty. This paper is the continuation of our previous study [4] that analysed the perception of Warsaw urban park quality reported by five groups of mobility-impaired respondents (n = 103). In the quantitative research [4], it was found that the main obstacle was the inappropriate pathway surface and that the main activity in the urban park was walking. Therefore, we looked more closely at the surfaces, outdoor amenities, the park’s functional program and the park’s functionality by taking a walk through the park with the study participants. The groups were designed to include people who use crutches, wheelchairs (manual or electric), or a cane.

The study aimed to identify the preferences and expectations of people with mobility difficulties for park quality. Acquiring knowledge about the needs of the respondents was the overarching goal.

The accessibility of city parks as perceived by people with mobility difficulties is rarely the subject of research, which is why the authors of the article intend to fill this gap. Studies concerning older users and their preferences for green spaces [56,57] are more common. We assume that this study will help set out the course of action for park modernization. We want to confirm the following hypotheses:

- hypothesis 1: Urban parks are not accessible to people with mobility difficulties.
- hypothesis 2: Despite physical barriers encountered in the parks, people still find non-physical benefits.

## 2. Materials and Methods

### 2.1. In-Depth and “Walk-and-Talk” Interviews

We conducted in-depth- and “walk-and-talk” interviews. The place and time chosen were convenient for each of the respondents, which allowed them to give longer and fuller statements. The interviews (13) were conducted with people who voluntary agreed to participate in the research. According to the literature, the minimum number of participants in an IDI should be related to the number of criteria characterizing the study sample (e.g., gender, age, work status) by multiplying each variable by three [58]. The main criteria in our study covered five variables: gender, age, disability description/description of a mobility difficulty, mobility aid, work status. Therefore, 13 interviews fulfilled the qualitative research requirements. Moreover, the designed size of the respondents group allowed for a more intimate contact and deeper answers to profound questions. The study sample was equal in terms of gender and varied in terms of mobility difficulty (motor or sight impairment). The first participants were indicated by the associations of people with disabilities, and subsequent contacts using the snowball method were indicated by earlier participants of the study. The scenario for the IDIs covered opened questions asked in a neutral way not to suggest answers. In case of insufficient information, researchers asked the respondents for details [57].

A pilot study conducted on the 5 June 2018 in front of the District Disability Assessment Team at 5 Gen. Andersa Street in Warsaw helped verify research assumptions, the accuracy of the questions and understanding. The full-scale research project was carried out from December 2018 to December 2020. The respondents indicated the time during which the “walk-and-talk” interview could be carried out.

The in-depth interview was the first stage of the study. In the second stage, the “walk-and-talk” interviews were conducted with the same respondents using the same questionnaire in two parks—the neighbourhood (local) park and the destination (super-local) park in Warsaw. The in-depth interviews took 29 h, and the field interviews took 38 h. Each interview lasted from 1 to 3 h. During the interviews, different infrastructure elements were discussed in the context of the park’s accessibility. The discussions formed the basis for identifying overarching themes and subthemes shared by the interviewees [58]. The respondents did not feel any discomfort during the walk-and-talk interview and had enough time to express their opinion. The “walk-and-talk” interviews were held at different times of day to report the most common site-specific experiences [59]. The “snowball” method was used to select an appropriate research sample [58,59].

The IDIs were helpful in the process of gaining trust of the participants, which is crucial in this kind of study. They also enabled compiling the gathered information into an overall picture of urban parks accessibility. Moreover, in-depth interviews also helped to prepare a moderator and participants for the “walk-and-talk” interviews and more detailed questions.

### 2.2. Interview Questionnaire

#### 2.2.1. In-Depth Interview Structure

The main thematic areas were based on four key features of the space formulated by Project for Public Spaces [45]: “accessibility and linkages”, “comfort and image”, “uses and activities” and “sociability” (Table 1) (Supplementary S1). Seven questions were in the use and activities section, three in access and linkages, seventeen referring to comfort and image and five for sociability. The part concerning respondents’ profile included age, gender, park visit frequency, park visit approach and architectural barriers in the park. This analysis tool is based on the placemaking approach to empower the local community in deciding on the shape of a public space, transform public spaces into vital places that highlight local assets, spur rejuvenation and serve common needs to help people judging any place, good or bad. The main focus is placed on social and cultural aspects and encouragement to take ownership of streets by the residents [60,61,62,63]. Suitability of the method was verified with the experience of Project For Public Spaces in evaluating public spaces all over the world [45]. The organization has worked as a research centre advising on public spaces in more than 3500 projects since 1975. The interview structure corresponded with the content of other commonly used tools, e.g., Irvine–Minnesota Inventory [64].

#### 2.2.2. Walk-and-Talk Interview Structure

The “walk-and-talk” interview questionnaire was created based on the in-depth interview questionnaire, formulated with more detailed questions. The primary thematic areas were divided into: “Uses and activities, “Access and linkages”, “Comfort and image” and “Sociability” (Supplementary S1) with changes and additions in the form of a “Park’s questionnaire” prepared by the authors of this paper according to Karolina Kaszuba’s “park evaluation questionnaire” [65]. Eighty nine criteria were created and then assigned to 12 subcategories classified into four categories as in in-depth-interview (the number of criteria from a given subcategory is given in parentheses): Access and linkages: connecting to the area (5), accessibility to the site (5); comfort and image: technical conditions (24), safety (18); legibility of marking (13), visual inspection (10), sanitary level (3) uses and activities: available activities (4), spaces used (3) and sociability: users (4) (Supplementary S1). The interviews were recorded and later coded and transcribed for further analysis.

#### 2.2.3. Interpretation

The study methods were inspired by an interpretative phenomenological approach to gain insight into individuals with disabilities experiences concerning city parks’ perceived accessibility. The aim of interpretative phenomenological analysis (IPA) was to explore how participants were making sense of their personal and social world [66] and how they perceived accessibility of green spaces in Warsaw, Poland. The analysis has its source in the theory of the phenomenology of place developed by Edward Relph [67,68,69]. Relph describes this persistent identity in terms of three components: (1) the place’s physical setting, (2) its activities, situations and events and (3) the individual and group meanings created through people’s experiences and intentions about that place [70].

### 2.3. Respondents’ Profile

#### 2.3.1. Study Sample

This continuation of the previous research [56] used the population of people who encounter architectural barriers or other difficulties in moving around the park space due to:

- motor or sensory disability (e.g., vision) requiring the use of a wheelchair, crutches, canes, etc., or
- the motor or sensory disability of the dependant requiring the use of a wheelchair, crutches, canes, etc.

Physical barriers while moving were considered, not the original cause of the limitation. The study participants’ profiles were related to the knowledge saturation concerning potential inconveniences in pedestrian mobility.

The respondents were thirteen Warsaw residents with mobility difficulties due to different factors. Participants (n = 13) ranged from 18 to 76 years of age (Table 2). There were seven females and six males in the sample. Participants were Polish. The interviews were conducted at the location chosen by the respondents (Żeromski Park or Wilanów Park). The meetings were contingent not on weather conditions, but on the respondents’ health condition on a particular day and time and their willingness to participate in the study. A small number of people (n = 3) refused to meet due to their health condition or reluctance to talk about their disability or difficulties.

#### 2.3.2. Ethical Considerations

As the study involved no invasive procedures, there was no need for ethical approval. Nevertheless, we fore-grounded ethical research practice in the design and conduct of the study including informed consent via opt-in (with ability to leave the study at any time for no specified reason) and personal data confidentiality (gender, age, type of mobility impairment and employment were used in reporting).

### 2.4. Study Site

**Figure 1:**
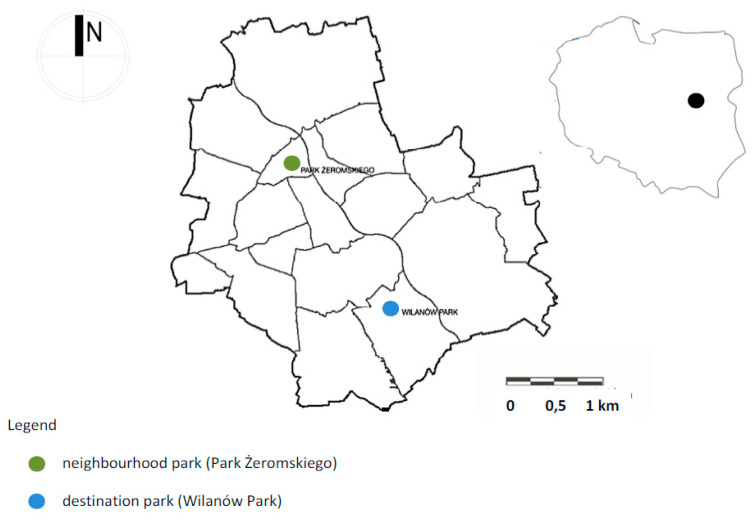
Location of the parks on the city map. Source: Processed by the Authors.

**Figure 2:**
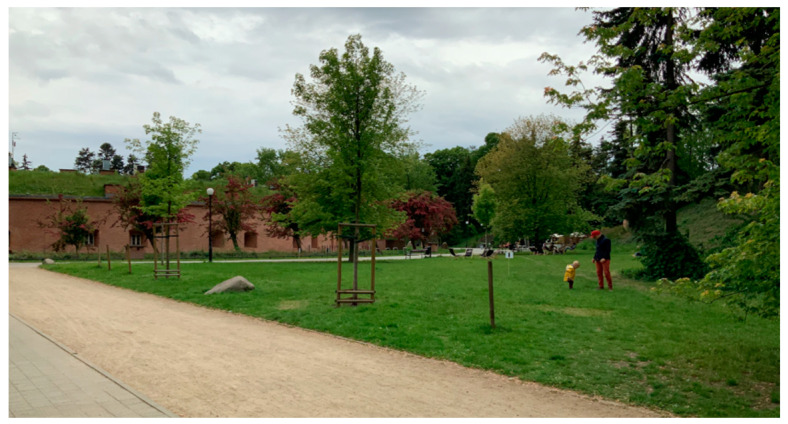
Żeromski Park in Warsaw (photo by Magdalena Wojnowska-Heciak).

**Figure 3:**
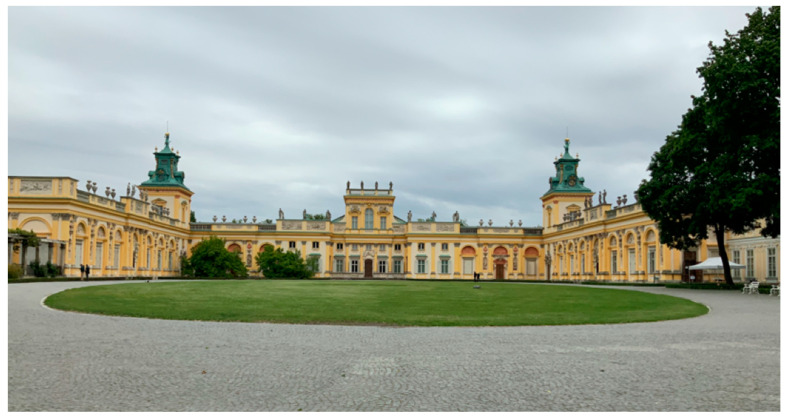
Main entrance to Wilanów Park in Warsaw (photo by Magdalena Wojnowska-Heciak).

As part of the study, two selected park facilities were assessed. These studies had a strictly defined field range, determined due to the availability of parks selected for the study. Due to the desire to learn about a wide range of barriers in recreation areas, one park of local importance and one of supralocal importance were selected. The first object, Żeromski Park in Warsaw, was indicated during a telephone conversation with an employee of the Department of Assistance to Persons with Disabilities from the Office of Social Assistance and Projects of the City of Warsaw. The second park was Łazienki Królewskie in Warsaw (the biggest historical urban park in Warsaw with 76 ha). During in-depth interviews and conversations about field interviews, the authors of the study were forced to switch to Wilanów in Warsaw due to the fear of problematic conditions in Łazienki Królewskie Park reported by the respondent. Therefore, smaller urban parks were selected for the study: Żeromski Park (6 ha) and Wilanów Park (24 ha) (Figure 1, Figure 2 and Figure 3). Warsaw boasts 79 parks with a total area of approx. 762 ha and varied historical background and importance. Many of them are listed historic parks and gardens [71].

Żeromski Park is located in Żoliborz district, at the Wilson Square, between Mickiewicza St. and Krasińskiego St. Its modernist style and location in a former fortress area assure unique advantages such as varied terrain, old forest, playground, a fountain with a sculpture and commemorative boulders. As a place of cultural heritage significance, the park was entered in the Cultural Heritage Register under no. 994A in 1980 [72]. The park is easily accessible by car and public transport (bus, tram, metro). Entrance to the park is free of charge.

Wilanów Park (Ogród w Wilanowie) is located in Wilanów district, approx. 10 km from the city centre, at the end of the historic Royal Route, leading from the Old Town with the Royal Castle to the Palace in Wilanów [73]. The park is located next to the original suburban residence of King John III Sobieski. It was established in the 2nd half of the 17th century on the common axis with the Palace of Wilanów. It covers 24 ha (45 ha together with Jezioro Wilanowskie (Lake Wilanowskie) and Kanał Sobieskiego (Sobieski Canal)). The 45-hectare complex includes buildings, gardens and parks of various styles: North Garden, Baroque Garden—divided into two terraces, Rose Garden, two landscape parks—the North and South, and the Orangery Garden. In 1965, the park was listed as a historical site in the Warsaw Province, and in 1994, it was recognized by the President of the Republic of Poland as one of the most important sites to Poland’s cultural heritage (Museum of King Jan III’s Palace at Wilanów, 2020). The paid parking area for passenger cars and coaches is located approximately 300 m from the Palace (behind the Post Office building). Wilanów can be reached from the city centre by public transport (buses) (Figure 2). The entrance fee for an adult is ca. 2 euro.

## 3. Results

### 3.1. Frequency of Park Visits

During the in-depth interviews, all respondents declared visiting the park several times a month. The majority of the respondents chose Żeromski Park for the “walk-and-talk” interview (n = 10). The park in Wilanów was known to all respondents, while some of them visited Żeromski Park for the first time. It is smaller than Wilanów Park and located closer to the city centre. Once they reached the park, they declared spending at least one hour there (Supplementary S2).

### 3.2. Access and Linkages

Despite various inconveniences during the in-depth interviews, respondents who used a wheelchair confirmed that they did not care whether they were to reach the local park near their residence or somewhere further—a destination park. Blind, partially sighted and older adults with disabilities preferred parks located closer to their homes to return fast if necessary. During the interviews, respondents gave their opinion on the park’s general accessibility (visibility and widths of entrances, steps). It was found that most of the participants encountered some difficulties and paid a lot of attention to the unfriendly infrastructure surrounding the parks. One of the respondents pointed out traffic lights turning red too quickly as a factor that hindered accessibility (Supplementary S2). 

Each “walk-and-talk“ interview started at the place reached by the respondent by car or public transport (car park, public transport stop) or—if the respondent walked to the park—from the agreed meeting spot located not more than 500 m from the entrance to the park. Most of the respondents did not live in the vicinity of the selected parks. Most often they used public transport. None of the respondents came to Wilanów Park on foot. In the case of Żeromski Park, only one person lived close enough to choose this way of getting there. Everyone declared that they did not care whether it was a park near their homes—a local park or a destination park (Supplementary S3: Table S1).

Respondents were also asked in situ if they experienced any difficulties or inconveniences in travelling to the park (Supplementary S3: Table S1). In the case of Wilanów, there was no problem in getting to the park for people arriving by public transport. A too small parking space was the problem for a person in a wheelchair who arrived by car. The most significant difficulty in getting to Żeromski Park was to get out of the underground (out-of-order lifts). Blind people had most difficulties in getting to the park. No tactile surfaces led to any gate in the park. In Żeromski Park, the lack of proper signs that the gate was closed and the entrance accessible only through a much narrower wicket gate made things difficult. The entrance to the park was also problematic from very busy Ludwika Mierosławskiego Street.

In this case, a crucial aspect is the location of the park in relation to pedestrian routes, public transport stops and parking spaces. During the study, the results of both types of interviews: IDI and “walk-and-talk” confirmed other researchers’ opinions—in shaping the space available to all, one should first focus on convenient transport options in the given area (Supplementary S2 and S3). All aspects of this subject are important, from low-floor vehicles to the surface of routes connecting bus stops with the park, so that pedestrian crossings are appropriately marked, and there is the presence of tactile information facilitating the location of the park by the blind and partially sighted, ending with the adaptation of the access surfaces. During this research, it was shown that transit connections or mode splits are often more important than the location of a given park in relation to the respondents’ place of residence, as they are able to travel to destination parks, if the public transport allows.

The hypothesis that urban parks are not accessible to people with a mobility difficulty has been confirmed in terms of inadequate quality of transit connections or insufficient number and size of entrances to parks.

### 3.3. Comfort and Image

According to the Project for Public Spaces tool, a space that is comfortable and looks inviting is likely to be successful. A sense of comfort includes perceptions about safety, cleanliness and the availability of places to sit. A lack of seating is the surprising downfall of many otherwise suitable places. The IDI study results revealed that the most common activity for people with mobility difficulties is walking in the park, despite the effort they put into moving around. Those with the most significant mobility problems signalled the need for amenities located directly at the park entrance. The majority of the respondents were able to take longer walks—up to one hour (Supplementary S2).

**Figure 4:**
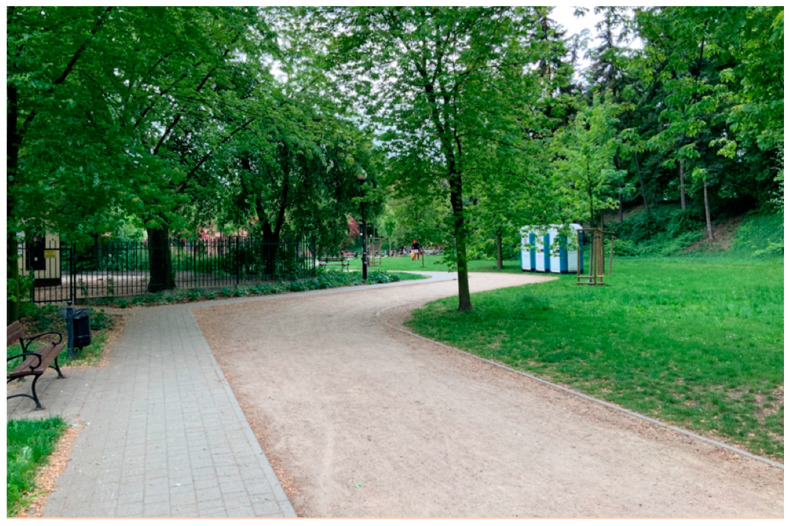
Gravel surface along the concrete walkway in Żeromski Park (photo by Magdalena Wojnowska-Heciak).

**Figure 5:**
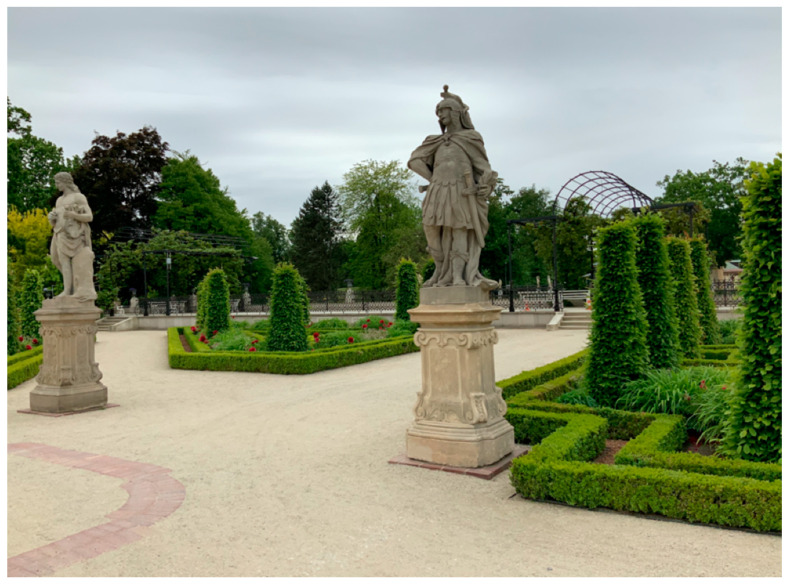
Gravel surfaces and stairs next to the Palace in Wilanów Park (photo by Magdalena Wojnowska-Heciak).

During the “walk-and-talk” interviews, they were asked about the comfort of moving around the park and the support for walking in the area (ramps, railings, tactile markings, keeping the optimal width, surface type). Asphalt surfaces were the best for people with mobility disabilities, and respondents with sensory impairments valued concrete surfaces the most (Figure 4 and Figure 5) (Supplementary S3: Table S2). In Wilanów Park, the respondents paid attention to uneven pavements and excessive lateral slope by the water reservoir, making it impossible for people in a wheelchair to pass. The pedestrian routes exceeding 8 percent grade also caused inconvenience. In Żeromski Park, inconveniences included uneven and diversified surfaces (Figure 2 and Figure 4), especially those made of gravel. In Wilanów, the slope of some alleys was the major obstacle (Supplementary S3: Table S2).

Respondents were asked about their perception of the outdoor furniture located in the park during the “walk-and-talk” interviews. According to them, resting places such as benches were important elements of the park. However, they were not always properly located. Benches mounted far away from the main paths were useless for some respondents. Those mounted on narrow pathways were difficult to pass. The participants noticed that there were not enough litter bins in the parks, and even if they were available, they were hardly accessible. Apart from places for passive rest, outdoor facilities should be adapted to people’s needs and abilities with mobility difficulties (Supplementary S3: Table S2). This problem was also highlighted during the IDIs (Supplementary S2). Another issue raised during the “walk-and-talk” interviews was whether the park’s infrastructure—outdoor furniture—is adequately adapted to the needs of people with mobility problems. In Wilanów Park, the respondents paid most attention to the lack of bays where benches could stand. In Żeromski Park, the only obstacle indicated by people in wheelchairs was the missing litter bins (Supplementary S3: Table S2). In most cases, people using a wheelchair declared that it is difficult to point out any inconvenience concerning locations or the number of benches as they have their seating with themselves. Partially sighted and blind people also did not point out any problem with outdoor furniture during the “walk-and-talk” interviews, whereas during the IDIs, they pointed out the problem of the lack of colour contrast between pavement surface and amenities.

In the IDIs, some of the respondents revealed that it was very important that information about the park’s layout was provided in the most suitable places, mainly at the entrances, and in some other parts inside the park (Supplementary S2). The information board in Wilanów Park was installed about 15 m from the park entrance. When asked about its legibility during the “walk-and-talk” interviews, respondents complained about difficulties with reading it and the absence of relevant information. In Żeromski Park, the information board is located right next to the park entrance. Nevertheless, it lacks basic information on adapted restrooms’ location or areas accessible to wheelchair users. The board was illegible for blind and partially sighted people. This problem was quite pronounced in Żeromski Park (Table S2).

Calm and peaceful places in the park, open for public use lawns and places near water reservoirs are only a few places indicated by the respondents as suitable for having some rest during park visits. In Wilanów Park, the most common places for relaxation were the areas near Wilanów Lake, and in Żeromski Park, these were the spaces near cafés. In Wilanów Park, adapted restrooms were unavailable. There was one accessible toilet in Żeromski Park. Restrooms in cafes and “toitoi”-type mini cabin toilets were not adapted (Supplementary S3: Table S2).

An essential aspect of the park is its safety. People with disabilities are especially exposed to unpleasant situations in urban spaces. However, the majority of respondents declared feeling safe and comfortable in the park, where they met people willing to help if necessary (Supplementary S3: Table S3).

The comfort and image section results prove that the pedestrian circulation is disturbed by many inconveniences (improper surface of the pathways, no comfortable rest places, lack of easily accessible restrooms). Hence, the predicted hypotheses that urban parks are not accessible to people with mobility difficulties is again confirmed by the results. In this section, the social factor’s role in the perception of urban park accessibility begins to emerge such as the feeling of security and safety and the possibility of receiving support from other park users. The hypothesis that in addition to physical elements (pedestrian surfaces and amenities in the park), diverse social needs of mobility impaired users are fulfilled during park visits, is confirmed.

### 3.4. Use and Activities

Land-use patterns were studied during the in-depth interviews (Supplementary S2). People differed in their choices. However, the differences did not result from the respondent profiles. Among the respondents, there were both working and non-working people. Most of those who had a job did not spend time in the nearby park despite living within a 15-min walking distance to it. Some of the respondents declared that they liked both types of parks (local and destination) (Supplementary S2 and S3).

During the “walk-and-talk” interviews, none of the respondents spent time only in the park of local importance (neighbourhood park) (Supplementary S3: Table S4). The amount of time spent in the park by city residents with mobility difficulties was also influenced by the number and quality of scheduled events and activities organized in the park (Supplementary S3: Table S4). Many participants preferred other parks, for example, those of supra-local importance (destination parks). People who did not work spent more time in the local (neighbourhood) park (Supplementary S3: Table S4). Respondents were asked about the feelings that usually accompanied them during their visits to the parks. All answers were positive (Supplementary S3: Table S4). Safe and comfortable, adequately marked and equipped with ramps and railed pavements was what the respondents of this study expected most (Supplementary S3: Table S4). Wheelchair users did not like gravel surfaces, claiming they were uncomfortable and unsuitable. Respondents agreed that the park lacked proper information boards or pathway markings (Supplementary S3: Table S4). Restrooms were either unavailable or were not adapted to their needs (Supplementary S3: Table S4).

The results illustrate the problem of inaccessibility of urban parks in some aspects, which confirms the first hypothesis.

### 3.5. Sociability 

Preferences for spending time in the park varied and were dependent on the individual needs of people. However, during the IDI, the majority of respondents indicated that they preferred to stay in a group (Supplementary S2). This was confirmed during the “walk-and-talk” interviews (Supplementary S3: Table S5). Asked about companions during their park visits, respondents replied differently; some preferred being alone (older adults); others enjoyed spending time with friends in a large group (young park-goers) (Supplementary S3: Table S5). In our study, respondents felt much more exposed to unpleasant situations and needed eye contact with other people in the distance. On the other hand, as we mentioned before in the comfort and image section (Table S3), most of the respondents believed that “people do not tend to attack the disabled” and generally felt safe in the park. Despite these inconveniences, the respondents declared feeling better after park visits (Supplementary S3: Table S5). It seems that the statement “I visit the park as often as I can” reflects the general trend. There is a strong belief among people that regular contact with quality, safe and inviting green spaces offers physical and mental health benefits, while limited or no access to parks creates a risk of social isolation, as confirmed in our studies (Supplementary S3: Table S5).

Despite limited usability, parks attracted people with disabilities for different reasons and in different ways. The prevailing motivation was a simple desire to enjoy outdoor nature and fresh air. Respondents were asked about their participation in any events organised in the two urban parks. All respondents were at Wilanów Park at least once and participated in the events offered there. For some of the respondents, it was the closest green area to their homes or work. Two of the respondents took part in the silent disco or “jewellery making course” in Żeromski Park.

During the study, blind respondents showed vivid interest in vegetation, shapes, smells, textures and sounds. They were also interested in the visual values of park areas, especially in the destination park. It was very important for them to be able to listen to accompanying persons or a stranger passer-by describing the surrounding nature. Certainly, a very important factor for these people is security, but rather in a social sense (few criminogenic factors, the presence of other people). The hypothesis that despite physical inconveniences, non-physical aspects (cultural events and social activities) are the motivation factors for a visit has found evidence in the results. The findings also indicate that accessibility means not only physical characteristics but also safety.

## 4. Discussion

### 4.1. Accessibility

In our study, the type of disability did not affect the perception of access-restricting factors such as insufficient adaptations, inadequate amenities, the lack of information or signage. The interesting finding was that the respondents refused to perform the “walk-and-talk” interview in Łazienki Królewskie Park because of its size and topography. In general, people with a mobility difficulty feel safer in smaller parks. Another element that needs to be studied in terms of accessibility is the park’s layout, its ambience and entrance fees. Entrance fees in Wilanów Park were not highlighted as a constraint by most of the respondents, which was interesting, considering that people with disabilities live modestly and pay attention to additional costs (even if there are discounts for people with a disability). Respondents did not mention the park style as a factor motivating them to visit. It seems that park aesthetics or fees do not have as strong effect on their satisfaction as the need for contact with natural environment and other people. Despite the long list (see Table S4) of unfriendly infrastructure, the respondents declared a positive approach to the park visit and better well-being. 

Considering the analysed variables (see Supplementary S2; Table S1), the study results revealed that despite inconveniences related to access and linkages or inadequate quality of pavement surfaces (see Supplementary S2; Tables S1 and S2), urban parks attract people with mobility difficulties (Supplementary S3: Table S4). The motivation for walking lies in the possibility of interacting with other people, socializing and participating in cultural events (Supplementary S3: Table S5). In general, people with mobility difficulties need to socialize, meet others and interact with them. They generally feel safe in urban parks and feel that in the case of emergency, there are some who will voluntarily help them (Supplementary S2; Table S3).

### 4.2. Physical Features of a Park 

One of the aspects discussed in detail with the respondents concerned park elements, such as surface, outdoor furniture, park equipment or information systems. The Ordinance of the Minister of Infrastructure, dated 12 April 2002, on technical conditions to be met by buildings and their location [74] also refers to the buildings’ surroundings and intends to guarantee a comfortable space for people with disabilities. The quality of the two parks in terms of physical infrastructure (pathway surface, stairs and ramps) meets the requirements set forth in the Ordinance to some extent. However, according to the respondents’ observations, the two urban parks are not properly adapted to their needs (Tables S1–S4). The respondents’ comments were similar to Kowalski’s [43] guidelines and the findings of Meshur [75]. Kowalski goes into more detail and mentions, for example, that when selecting the surface material, glossy surfaces should be avoided as they can confuse those visually impaired by glare. However, certain elements Kowalski finds acceptable, such as gravel pathways, were reported as uncomfortable by those with a mobility impairment.

All respondents indicated the pathway surface as the one that caused difficulties. Too slippery, uneven or sand and gravel paths were indicated as one of the main barriers in parks. This finding is quite surprising for the authors who, while actively working in the design field, preferred stabilized sand and gravel surfaces in green areas as nature-friendly and water-permeable [24,76,77,78]. This pavement surface characteristic is not covered in the guidelines of universal design though this should serve as a direction in reconciling the needs of all users [74]. The same situation occurred with the size of the park. Respondents reported difficulty in finding their way or just the entrance to the park. For this reason, they preferred smaller parks. The tools for enhancing accessibility could include ICT solutions [79] or dedicated wayfinding applications and navigation services with support functions, such as an extension in the form of CityGML ADE or Route Accessibility Index [79,80].

### 4.3. Non-Physical Benefits of a Park Visit

Green spaces need to be accessible for all users to benefit them physiologically, psychologically and socially, offering all available opportunities [32,81,82,83]. Urban green and blue spaces are still poorly considered in planning [41]. It is important also because people with a disability report low levels of physical activity [84] and are more likely to rate their health as poor [34].

In our study, people with a mobility problem, despite reported difficulties to get there, seemed to visit a city park as often as possible. According to Wojnowska-Heciak [19], Warsaw residents prefer to spend their free time outdoors in the city park as the first choice and at the Vistula River as the second choice at least once a month. The scores show that transport accessibility is a relevant factor. Other researchers also confirm that people with a disability are more frequent park visitors than others [2,85]. Our study results revealed that local parks are preferred by older adults, but in general, people with a disability do not choose one park over another. They like to learning new things and sometimes change usual destinations. This was also confirmed in their preferences showing that after a visit to Wilanów Park, they planned a trip to Żeromski Park.

The preferred forms of recreation among people with a disability are meeting relatives and friends, spending free time at home or on a garden lot, but it is walking [19] and social activity that increase integration and inclusion [86]. Corazon [37] suggested that interpersonal contacts with users of green spaces can be either a potential constraint (strengthens feelings of exclusion) or a stimulus to go out with positive feelings [37]. Godbey et al. [87] suggested that personal reasons are one of the key constraints for visiting green spaces by visitors having no mobility difficulties. Our respondents reported a feeling of satisfaction or mental rest after a visit to the park, even when feeling tired afterwards. Despite the weak health of some of them, the visit to the park proved to be beneficial.

Feeling safe was another important aspect [37]. As revealed by Corazon [37] and Darcy, Lock & Taylor [88] (2017), fear of personal safety was the only major intrapersonal constraint [37,88]. In our study, respondents reported feeling relatively safe in the parks. Even if the assistance offered by passers-by was overly intrusive at times, social factors seem to be crucial for the respondents’ sense of security; it can be either staying within sight or among a group of people. Moreover, a park or just natural environment can be a safe place for people with disabilities because, as demonstrated in e.g., [87], being among trees and other vegetation reduces aggression and crime level among visitors. Our respondents confirmed these findings.

Both interview types, IDI and “walk-and-talk”, allowed gathering complementary information [88]. At the same time, the contact established during IDI positively affected freedom of expression and openness during the “walk-and-talk” interviews. Minor differences appeared in the case of the in-depth-interviews (Supplementary S2). Unlike in the “walk-and-talk” interviews where the focus was on walking, in the in-depth-interviews, a general social dimension (meetings with friends, playing with children) was added as equally important. It seems that once the first need is satisfied, a walk in the park, meetings are the second most important activities. During the in-depth interviews, the respondents most often declared spending time in local parks, whereas during the “walk-and-talk” sessions, they indicated destination parks as preferred.

During in-depth interviews, the respondents declared the use of attractions and equipment offered by parks. In the case of “walk-and-talk” interviews, 11/13 respondents denied using them. This discrepancy can be explained by referring to question 16 in the “walk-and-talk” interviews, where the majority declared that they would use extra infrastructure if it were available in the park (Supplementary S1–S3).

The research hypotheses were confirmed. Although the potential of urban parks for people with a mobility difficulty is huge, access to safe, welcoming public spaces remains limited. Further, landscape architects and city policy makers must keep in mind that the concept of accessibility extends over both physical elements and diverse social needs.

### 4.4. Study Limitations

The respondents varied in terms of age and mobility problems but constituted a group with similar urban park accessibility needs. Generalization of the results was difficult due to the sample size and research type (qualitative).

Due to the small research sample and already in the process of comparing data from in-depth and “walk-and-talk” interviews, we did not observe significant differences in the participants’ responses with motor and sight disabilities. There is potential for further research based on the IPA with more numerous participant samples. It is worth noting that blind or visually impaired people paid more attention to the sensory experiences they experienced in the park: the role of nature. In contrast, people with motor disabilities focused on the physical aspects of the park. Park users who used wheelchairs confirmed that they could reach the local park near their residence or somewhere further, while blind and partially sighted preferred parks closer to their homes. Wheelchair users more often declared that they walk in the park, while blind and partially sighted said they meet with friends. Wheelchair users more often use public transport to reach the park, while blind and partially sighted people do not. However, it was shown in this study that, in general, similar barriers affected all respondents to varying degrees, regardless of mobility problems.

The pandemic prolonged the time needed to collect the interviews.

## 5. Conclusions

In the 21st century, people with a disability still face problems when trying to access public spaces. The findings of this research, when displayed in the media, could be used to attract broader public attention. It is not only the park’s infrastructure that determines the quality of the space; social aspects play an important role here as well. More focus should be given to encouraging individuals with mobility difficulties to visit parks and to organize more events dedicated to social interaction.

Mobility difficulty should not prevent anyone from getting outside to enjoy fresh air, improve self-esteem and mood and counteract exclusion and depression. The research should concentrate on further exploration of public space accessibility improvement, bearing in mind the needs of persons struggling with mobility issues. The biggest challenge seems to be building the self-confidence, dignity and social equality of all members of society. Our research, focused on finding best solutions for the comfort of walking and moving in urban parks, should be continued to extend over all social groups (universal design). The application of our accessibility promoting findings is important from the point of view of public policy and funding priorities.

## Figures and Tables

**Table 1:** Key attributes and associated measurement tools used to analyse the perception of park accessibility of people with disabilities.

Key Attributes and Measurement Tools	Description of the Aspects Raised by the Respondents
Access and linkages	
Transport dimension Pedestrian activity	Distance from the place of residence to the park Means of transport necessary to reach the park/modes of transport Perception of convenience and accessibility
Parking usage pattern	Distance to the park entrance from parking lots or bus
Comfort and image	
Building conditions Crime statistics	Ease and comfort of moving around the areas Whether entrances to the park are well marked Quality of infrastructure in the park (benches, litter bins, toilets) The presence of facilities for the disabled Access to information about events in the park Health benefits after park visit Safety in the park
Uses and activities	
Land-use patterns Property values	Activities undertaken in the park Attractions to stimulate park uses
Sociability	
Social networks	Individually or with a group of friends Meeting new people in the park
Volunteerism	Whether other people are helpful in overcoming the physical barriers in the park

Source: Processed by the Authors.

**Table 2:** Respondent’s profile.

Participant Code	Gender	Age	Disability Description	Mobility Aids	Work Status
M1	M	50–59	Lack of sensation in the legs	Wheelchair	Employed
F1	F	30–39	Multiple sclerosis, paresis of upper limbs, lack of sensation in the legs	Wheelchair	Employed
M2	M	30–39	Cerebral palsy	Crutches	Unemployed
F2	F	<60 (76)	Age-related walking difficulties	Walker	Retired
M4	M	<60 (73)	Blind	Cane	Retired
F4	F	50–59	Blind	Cane	Employed
M5	M	18–29	Partially sighted	Cane	Student
M6	M	<60 (67)	Partially sighted	Cane	Retired
M3	M	18–29	A seriously injured person after a car accident	Electric Wheelchair, Crutches	Unemployed
F3	K	18–29	Progressive lack of feeling in the legs	Electric wheelchair	Employed (online working)
F5	K	50–59	An injured person after a car accident	Wheelchair	Employed
F6	K	30–39	Partially sighted	Canes	Employed
M7	M	40–49	Blind	Canes	Employed

Source: Processed by the Authors.

## Data Availability

Not applicable.

## Supplementary Materials

The following supporting information can be downloaded at: https://www.mdpi.com/article/10.3390/ijerph19042018/s1, Supplementary S1—In-depth interview questionnaire & “Walk-and-talk” interview questionnaire; Supplementary S2—“IDI & walk-and-talk interviews results”; Supplementary S3—Selected quotes from “walk-and-talk” interviews Tables S1–S5.
