# Supplementary material for: Urban Parks as Perceived by City Residents with Mobility Difficulties: A Qualitative Study with In-Depth Interviews
Source: Int J Environ Res Public Health. 2022 Feb 11;19(4):2018. doi: 10.3390/ijerph19042018 (PMC8871772; doi:10.3390/ijerph19042018)
Supplement: Supplementary file 1 [file ijerph-19-02018-s001.zip › Supplementary 1_interviews_structure.pdf]

International Journal of
*Environmental Research and Public Health*

MDPI

# Supplementary 1.

# In-depth interview questionnaire

USES AND ACTIVITIES:

1. Do you often spend time in the park?
2. Do you prefer to spend time in a supra-local or local park?
3. How long do you spend in the park?
4. How do you rest in the park and how would you prefer to rest?
5. Why do you spend time in the park?
6. Do you use the attractions offered by the park (outdoor gyms, performances, board games)?
7. What events / attractions would make you start spending more time in the Park?

ACCESS AND LINKAGES:

1. Is there a park near your place of residence, how far is it?
2. Do you use public transport to get to the park? If not, how do you get to the park?
3. What do you think about accessibility to the park?

COMFORT AND IMAGE:

1. Are the park entrances properly marked?
2. Do the parks have signs to make it easier to navigate?
3. Is it easy for you to find your way around the park / move around it?
4. Is the surface in the parks adapted for people with disabilities / mobility difficulties?
5. Is the appropriate infrastructure used in the parks to facilitate movement? (ramps, surface color, suitable surface). If so, does it fulfill its functions?
6. Do you think there are many green areas in the parks that are inaccessible to people with disabilities / mobility difficulties?
7. Do you think there are places in the park where you could relax freely? If so, are there enough of them?
8. In your opinion, is the park equipment (such as: benches, outdoor gyms, etc.) properly marked and "friendly" for people with disabilities?
9. In your opinion, do you think there are toilets in the park that are suitably adapted to the needs of the disabled?
10. What kind of barriers in the park do you have to deal with?
11. What, in your opinion, is necessary in the "Park without barriers"?
12. If the park was more accessible, would you spend more time in it?
13. If there were aids in the park intended for people with your disability / reduced mobility, would you use it?
14. Would you like to have access to information about events that are organized in parks in your area?
15. Do you feel comfortable in the park?
16. How do you feel after your stay in the park?
17. Do you feel safe during your stay in the park?

SOCIABILITY:

1. Do you like to spend time in the park alone or with friends?
2. Do you spend your time alone or in a group in your everyday life?
3. Do you establish a conversation, new acquaintances in public space?
4. Does the park meet with the offer of help from other users in overcoming obstacles / difficulties?
5. Are the people in the park helpful and friendly to you?

# "Walk-and-talk" interview questionnaire

## PART A - PARK'S QUESTIONNAIRE

1. Do you like to spend your free time in the park?

□ Definitely yes
□ Probably yes
□ It's hard to say
□ Not really
□ Definitely not

International Journal of
*Environmental Research and Public Health*

MDPI

2. How often do you visit the park?
□ Every day
□ Several times a week
□ Several times a month
□ Several (up to a dozen) times a year
□ I don't visit parks
3. How much time do you most often spend in the park?
□ Up to 30 minutes
□ About 1 hour
□ About 2-3 hours
□ More than 3 hours
4. If you visit the park, what activities do you most often do?
□ Walk
□ Conversation
□ Observing your surroundings
□ Reading a book
□ Using an outdoor gym
□ Other ……………………………………………………………………………………
5. What would make you spend your time in the park more often?
□ More benches / seats
□ Organized meetings / events in the park
□ Opportunity to meet new people
□ Possibility to meet friends
□ Better road surface for easier movement
□ Easy access to the park
□ Attractive plant compositions
□ Other ……………………………………………………………………………………
6. Do you prefer to spend time in a park of supralocal significance (eg Łazienki Królewskie, Wilanów Palace and Park Complex) or a local park (eg parks close to your place of residence, pocket parks)?
□ I spend my time only in parks of supra-local importance
□ I spend time in both types of parks, but more often of supra-local importance
□ I only spend time in parks of local importance
□ I spend time in both types of parks, but more often of local importance
□ I spend the same amount of time in supra-local and local parks
7. To what extent, on a scale of 1-5, is the accessibility of the park important to you? Please mark the appropriate amount on the scale. (1 is very little important and 5 very important)?
1 2 3 4 5
very little important □ □ □ □ □ very important
8. Please indicate to what extent, in your opinion, the following parks are available (1 point is unavailable, and 5 points are completely accessible to disabled people). 1 2 3 4 5
Destination parks □ □ □ □ □
Local parks □ □ □ □ □
9. Do you encounter architectural barriers in the park (inadequate surface, no benches, no ramps, etc.)?
□ Definitely yes
□ Probably yes
□ It's hard to say
□ Not really
□ Definitely not
10. What kind of difficulties are the most onerous for you? (Please select a maximum of 3 answers)?
□ Inadequate pavement
□ Inadequate marking of park entrances / exits
□ Too few benches / seats
□ Lack of appropriate park marking (possibility of getting lost in it)
□ Lack of an appropriate park program (nothing interesting is happening there)
□ No sense of security
□ Difficult access to the park (no elevators from underground passages, unmarked pedestrian crossings, etc.)
□ Lack of toilets adapted to the needs of disabled people.
□ There are no ramps near the stairs or an alternative road suitable for people with reduced mobility
□ No handrails on the stairs
□ The steps or curbs are too high
□ Inadequate sidewalk width
□ Other: obstacles in the middle of the path, no color contrast of the equipment
11. In your opinion, is the surface in the park alleys properly adapted to moving around?

□ Definitely yes
□ Probably yes
□ It's hard to say
□ Not really
□ Definitely not
12. Do you think that the park alleys are wide enough to move around the park comfortably?
□ Definitely yes
□ Probably yes
□ It's hard to say
□ Not really
□ Definitely not
13. Do you think there are enough places for rest in the parks?
□ Definitely yes
□ Probably yes
□ It's hard to say
□ Not really
□ Definitely not
14. Do you use the attractions offered by the park or park equipment (outdoor gym, organized meetings, playgrounds for adults, board games, etc.)?
□ Definitely yes
□ Probably yes
□ It's hard to say
□ Not really
□ Definitely not
15. Do you use mobile applications that help you navigate in public space? If you use such help, please mark the last two answers and in the point "Other answer" enter their name.
□ No, and I won't use them even if I knew about them
□ No, but if I had known about them, I would love to use them
□ Yes, I have
□ Other: no
16. Do you use auxiliary materials available in the parks (maps, models, navigators, etc.)? If you use such help, please mark the last two answers
□ No, and I will not use it even if it is available
□ No, but if they were available I would love to use them
□ Yes, I have
□ Other: no

RESPONDENT'S PROFILE:

1. Gender
□ Female
□ Male
2. What is your age?
□ 18 -29 years
□ 30-39 years
□ 40 - 49 years
□ 50 - 59
□ <60
3. Education
□ Primary
□ Vocational
□ High school
□ University degree
□ Student
4. What makes moving difficult for you?
□ I am a wheelchair or electric wheelchair user
□ I am a person with a walking disability, on a cane, on crutches
□ I am blind myself
□ I am a visually impaired person

International Journal of Environmental Research and Public Health

MDPI

□ I am the child's parent / carer

## PART B – DETAILED STUDIES PERFORMED *IN SITU* BASED ON IN-DEPTH-INTERVIEW

ACCESS AND LINKAGES:

1. Is this park close to your place of residence?
2. Did you use public transport to get to the park? If not, how do you get to the Park?
3. Did you find it difficult to get to the park?
4. What do you think about accessibility to the park?

COMFORT AND IMAGE:

1. Are the park entrances properly marked?
2. Does the park have signs to help you navigate through it?
3. Is there an information board in the park? If so, is it legible to you?
4. Is it easy for you to find your way around the park and to move around it?
5. Is the surface in the park suitable for people with reduced mobility?
6. Is there an appropriate infrastructure in the park to facilitate movement. (ramps?, surface color, appropriate surface?) If so, does it meet your requirements?
7. In your opinion, are there areas in the park that are inaccessible to people with walking difficulties?
8. Are you able to indicate a place where you could rest freely?
9. In your opinion, is the park equipment (such as: benches, outdoor gyms, etc.) properly marked and "friendly" for people with disabilities?
10. In your opinion, are there toilets in the park that are appropriately adapted to the needs of the disabled?
11. Are there any barriers in the park? If so, what are they?
12. What would you change in this park?
13. Did this visit encourage you to visit the park again?
14. Do you feel comfortable in this park?
15. How do you feel after your stay in the park?

USES AND ACTIVITES:

1. What would you change in the park?

SOCIABILITY:

1. Have you ever been to this park? If so, what impression did this Park make on you the first time?
2. Have you ever heard about this park? Do you know about the events and attractions in this park?
