# Supplementary material for: Urban Parks as Perceived by City Residents with Mobility Difficulties: A Qualitative Study with In-Depth Interviews
Source: Int J Environ Res Public Health. 2022 Feb 11;19(4):2018. doi: 10.3390/ijerph19042018 (PMC8871772; doi:10.3390/ijerph19042018)
Supplement: Supplementary file 1 [file ijerph-19-02018-s001.zip › Supplementary_3_tables_S1_S5.pdf]

International Journal of
Environmental Research and Public Health

MDPI

Supplementary 3

SELECTED QUOTES FROM "WALK-AND-TALK" INTERVIEWS

3.1. Access and linkages

Table S1. Pedestrian activity, Traffic data, Mode Splits.

Variable	Respondent's profile	Responses
Pedestrian activity – park's accessibility from the outside		
	F4	*Pedestrian crossings without lights, no indication in the pavement where the park gate is located, no information at the stop where what is located. When I go to the park for the first time, I need "a navigator", only after the second, third time, depending on the difficulty, I can go alone.*
	M5	*It's very difficult to get into the park, it's often just too high a threshold, a step.*
	M1	*Sometimes there is a footbridge and an elevator in the park and the elevator is always broken. If it wasn't for the footbridge, I wouldn't have a way to go (...); I want to say that there are many barriers that a "wheelchair driver" has to cope with: a bad surface leading to a park, some slightly too high steps, but if amenities are out of order all the time, it's not help, and the worst of the barriers*
	M3	*Accessibility is a flat entry*

International Journal of
Environmental Research and Public Health

MDPI

Traffic data, Mode Splits, Pedestrian activity		
Wilanów Park	*M1*	*All public vehicles are low-floor, so it is easy to get there.*
Park Żeromskiego	*F2*	*It's confusing. I sometimes stay in this park even though I don't live near it and it's different every time. It's probably because of the time of day, but the information is nowhere to be found. That's why I always go through the wicket gate, even when the main gate is not closed.*

Source: Processed by the Authors.

3.2. Comfort and image

Table S2. Building conditions – stairs, surfaces, infrastructure, information board.

Variable	Respondent's profile	Responses
Building conditions – stairs		
	M2	*First of all, the stairs, they're very slippery, improperly sized, you have nowhere to lean back and feel confident.*
	M5	*The lack of yellow markings on the beginning of the stairs and the end of the staircase make me unable to see them.*
Building conditions – surfaces		
	M1	*These water-permeable surfaces stick terribly to the wheels when it rains lightly, it's very difficult to roll a wheelchair over something like this*
	M4	*Much more comfortable are the concrete surfaces, yes, the fall is much more painful on*

International Journal of Environmental Research and Public Health

MDPI

F3, M7	*them, but there are no pebbles under the cane. On an even surface, it is easier to catch some unevenness. On a gravel surface, you have to watch out for so many things. The surface is the greatest challenge in the park, the width of the alleys is fine. The best is a smooth surface. The park managers should remove branches, stones, sand and gravel from the surface. Often after staying in the park I feel "shaky". the color of the surface is also important, the worst are protruding obstacles, sometimes big stones are located near the path merge with the path, there must be a contrast between the color of the surface and the edge*
Building conditions – the pathways on the slopes	
M1	*It is very difficult to climb this hill in Żeromski Park.*
Building conditions – infrastructure: benches, litter bins, information board	
F2	*For the elderly, a backrest is necessary to relieve strain on the spine and a hand rest to get up. And the seat mustn't be too low*
F1	*The trash bins are often simply inaccessible to a sitting person. You have to pull up on the backrest of the wheelchair and throw things in*
M4	*I use the outdoor gyms quite frequently. It would be nice if they were more accessible, for example, by using small protrusions to show what you can exercise on them or what parts of the body work, but rather more graphically than using Braille. In fact, it is used by people*

International Journal of
Environmental Research and Public Health

MDPI

		who have not seen since birth, which means some 20 percent of blind people.
	M6	*The big problem is the signs when they're too low. In my park by the house, that's just the way it is, before I learned where it stands, it hurt to remind me of*
	M5	*Hurdles, fences of all kinds are a big risk, sharp elements at the height of the hand of such barriers, then "you're really in a jam"*
	F4	*Walking on the sidewalk, I'm not expecting a bench. The cane can't always show that this bench stands in my way. Then it is very unpleasant to hit my knees on a bench*
Building conditions – information board		
Wilanów Park – location of the information board in the park	M1	*The board is too high and has too small letters to read I have to get very high, I would have to move away, which makes the letters unreadable*
	M4	*The board is not suitable for blind people, but I know that in the palace you can ask for tactile plans specially designed for blind and partially sighted people. We can approach them. (...) These plans are very well and legibly made, but first someone has to explain to me the basis for reading such a plan or legend.*
Building conditions – toilets		

International Journal of
Environmental Research and Public Health

MDPI

	M1	*I'd have to ask someone for help, and it's quite embarrassing for me and for this person*
	F1	*The toilet is not adapted to the abilities of disabled people"*
	F3	*The toilets may be suitable for wheelchair users but not electric wheelchair users*

Source: Processed by the Authors.

Table S3. Crime statistics.

Variable	Respondents profile	Responses
Crime statistics		
	M1	*I feel good in the park, whether night or day, yes, I'm in a wheelchair, but it also has its advantages. People aren't likely to attack the handicapped.*
	M4	*Everyone is nice and kind. When I ask for directions, there will always be someone to help me, and sometimes even guide me. It happens this help is too "intense" and therefore dangerous. I often have such situations at pedestrian crossings or on public transport. Strangers come up to me, grab me under my arm and walk. I honestly have no idea where. Once I found myself on the wrong bus, which I boarded because somebody wanted to help me. It's important to help, but not force it, because then it's not help anymore.*
	F1	*People tend to be helpful. Yet sometimes a disabled person who faces a challenge wants to*

International Journal of
Environmental Research and Public Health

	cope on their own, and that's when five people insist on helping me to cross a curb. My only point is that you should ask first.

Source: Processed by the Authors.

3.3. Uses and activities

Table S4. Land-use patterns.

Land-use patterns – Motivation to a park visit	
M1	*I go to the park every day, although it takes me fifteen minutes to go from point "A" to point "B"... I enjoy sitting in the park and reading a book especially in winter to enjoy the sunlight and its natural warmth.*
M5	*I go to the park a few times a month, depending on the amount of time I have, but when I go to the park, I spend a lot of time in it, even up to three hours.*
M2	*When I go to some organized event, I spend much more time there than usual; after a walk I feel like I haven't wasted that time.*
F2	*If I can talk to somebody, I would spend more time in the park. But it's important that I feel comfortable. Nobody wants to feel inferior.*
M3	*Attractive plant compositions encourage me to stay in the park.*<br>*I feel relaxed after being in the park.*

International Journal of
Environmental Research and Public Health

MDPI

F4	*The attractiveness of the place is increased by additional elements in the park like: binoculars, a bench of "happiness", a bell…*
Land-use patterns – Well-being after the park visit	
M1	*Even though I got a little tired of these hills, I'm mentally rested.*
M6	*Very positively, Wilanów is my favorite park.*
M5	*I rarely walk in the park, but I feel good when I am there.*
Land-use patterns - Shared activities	
M5	*I often use the outdoor gym, I am very active physically. I run a lot or do nordic walking, of course with the help of an accompanying person. Sometimes, however, spending time in the park is for socializing: I have a large group of friends. From May to October we meet in the park. You can then actively spend time with your friends and there's a reason to leave your room.*
F4	*I like being among trees and tall grass. I like the noise they make. Sometimes I even ask people passing by to tell me what colour the tree is. It's a win-win situation, I think. I benefit from such an interaction, because I can imagine it better, and this person too, because probably if I hadn't asked them, they wouldn't even have paid attention to this plant.*
M1	*I like to sit in the park and read a book especially in winter to catch some sunlight and its natural*

International Journal of
Environmental Research and Public Health

MDPI

		warmth. A walk in the park in winter is the best way to give your body some vitamin D.

Source: Processed by the Authors.

3.4. Sociability

Table S5. Social network.

Variable	Respondent's profile	Responses
Social networks		
	M1	*I'm spending time in the park alone, I'm getting older and I need more and more peace.*
	M5	*I spend time in the park with my friends, in a large group, often someone will join in. That's how you meet really interesting people.*
	F4	*I like having a few friends with me, we often go for a walk in a park.*
	F2	*I was at the exhibition of park rose species exhibition.*
	M4	*I take part in events dedicated to blind people. Then you can learn interesting things about the park.*

Source: Processed by the Authors.
